# Neuroprotective Effects of Genome-Edited Human iPS Cell-Derived Neural Stem/Progenitor Cells on Traumatic Brain Injury

**DOI:** 10.1093/stmcls/sxad028

**Published:** 2023-04-08

**Authors:** Ryotaro Imai, Ryota Tamura, Masahiro Yo, Mizuto Sato, Mariko Fukumura, Kento Takahara, Yoshitaka Kase, Hideyuki Okano, Masahiro Toda

**Affiliations:** Department of Neurosurgery, Keio University School of Medicine, Shinjuku-ku, Tokyo, Japan; Department of Neurosurgery, Keio University School of Medicine, Shinjuku-ku, Tokyo, Japan; Laboratory for Cell Function and Dynamics, RIKEN Center for Brain Science, Wako, Saitama, Japan; Department of Neurosurgery, Keio University School of Medicine, Shinjuku-ku, Tokyo, Japan; Department of Neurosurgery, Keio University School of Medicine, Shinjuku-ku, Tokyo, Japan; Department of Neurosurgery, Keio University School of Medicine, Shinjuku-ku, Tokyo, Japan; Department of Physiology, Keio University School of Medicine, Shinjuku-ku, Tokyo, Japan; Department of Physiology, Keio University School of Medicine, Shinjuku-ku, Tokyo, Japan; Department of Neurosurgery, Keio University School of Medicine, Shinjuku-ku, Tokyo, Japan

**Keywords:** CRISPR/Cas9, genome editing, iPS cell, neural stem/progenitor cell, traumatic brain injury

## Abstract

Despite developing neurosurgical procedures, few treatment options have achieved functional recovery from traumatic brain injury (TBI). Neural stem/progenitor cells (NS/PCs) may produce a long-term effect on neurological recovery. Although induced pluripotent stem cells (iPSCs) can overcome ethical and practical issues of human embryonic or fetal-derived tissues in clinical applications, the tumorigenicity of iPSC-derived populations remains an obstacle to their safe use in regenerative medicine. We herein established a novel treatment strategy for TBI using iPSCs expressing the enzyme-prodrug gene yeast cytosine deaminase-uracil phosphoribosyl transferase (yCD-UPRT). NS/PCs derived from human iPSCs displayed stable and high transgene expression of yCD-UPRT following CRISPR/Cas9-mediated genome editing. In vivo bioluminescent imaging and histopathological analysis demonstrated that NS/PCs concentrated around the damaged cortex of the TBI mouse model. During the subacute phase, performances in both beam walking test and accelerating rotarod test were significantly improved in the treatment group transplanted with genome-edited iPSC-derived NS/PCs compared with the control group. The injury area visualized by extravasation of Evans blue was smaller in the treatment group compared with the control group, suggesting the prevention of secondary brain injury. During the chronic phase, cerebral atrophy and ventricle enlargement were significantly less evident in the treatment group. Furthermore, after 5-fluorocytosine (5-FC) administration, 5-fluorouracil converted from 5-FC selectively eliminated undifferentiated NS/PCs while preserving the adjacent neuronal structures. NS/PCs expressing yCD-UPRT can be applied for safe regenerative medicine without the concern for tumorigenesis.

## Graphical abstract

**Graphical Abstract:**
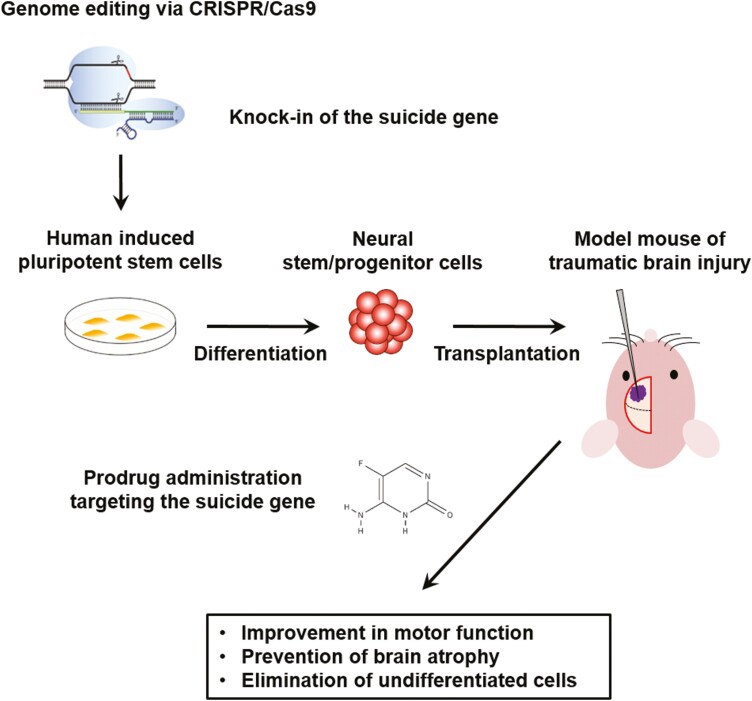

**Significance Statement**

Research on cell transplantation therapy for the central nervous system is rapidly advancing. Among the various cell types, induced pluripotent stem cells can overcome ethical and practical issues, while their potential to form tumors remains a safety concern. We established genome-edited induced pluripotent stem cells containing a safeguard, a suicide gene, to eliminate undifferentiated cells after the transplantation. We also demonstrated that neural stem/progenitor cell transplantation effectively improved motor function and prevented brain atrophy in the mouse model of traumatic brain injury. The present concepts can be applied not only to traumatic brain injury but also to strokes or other neurological disorders.

## Introduction

Traumatic brain injury (TBI) consists of hemorrhage, cerebral infarction, necrosis, and edema.^1^ Despite developing neurosurgical procedures, few treatment options have achieved functional recovery from TBI.^2^ Most patients cannot fully recover because regeneration is limited in the adult brain. Therefore, TBI is a continuous public health concern with limited treatment options.

Cell transplantation therapies employing a wide range of stem cells have recently attracted attention. Mesenchymal stem cells (MSCs) can easily be harvested from bone marrow and adipose tissue. Transplantation-mediated trophic support and immune regulation are the main mechanisms by which MSCs support the repair of damaged brain tissue.^3^ According to the latest research, bone marrow-derived MSCs improve motor deficits after TBI.^4^ However, the disadvantage of MSCs is that they cannot efficiently differentiate into stable neural tissue, meaning functional recovery primarily depends on the secretion of neurotrophic factors during the acute phase.^5^

Neural stem/progenitor cells (NS/PCs), however, can differentiate into neurons and glial cells to produce long-term treatment effects and functional recovery by repairing neural structures after brain injury.^6^ Some clinical attempts have been made to apply human NS/PCs derived from fetal cortical brain tissue or spinal cord to treat ischemic stroke.^7,8^ However, along with the accompanying ethical issues, NS/PCs are difficult to obtain from human embryos. Induced pluripotent stem cells (iPSCs) have the potential to overcome ethical and practical issues for clinical application,^9^ while tumorigenicity remains a major concern for the safety of iPSC-based regenerative medicine.^10-13^

Gene-directed enzyme prodrug therapy is an attractive approach to prevent potential tumorigenesis.^14,15^ In general, induction of stable constitutive transgene expression by viral vectors is difficult in human iPSCs (hiPSCs).^16,17^ Recently, we demonstrated *ACTB* as the most appropriate gene locus to achieve stable constitutive transgene expression in hiPSCs via CRISPR/Cas9-mediated genome editing.^18^ Herein, we established a novel gene therapy for TBI using NS/PCs derived from human genome-edited iPSCs expressing an enzyme-prodrug gene.

## Materials and Methods

### Cell Culture and CRISPR/Cas9-Mediated Genome Editing

**Figure 1.**
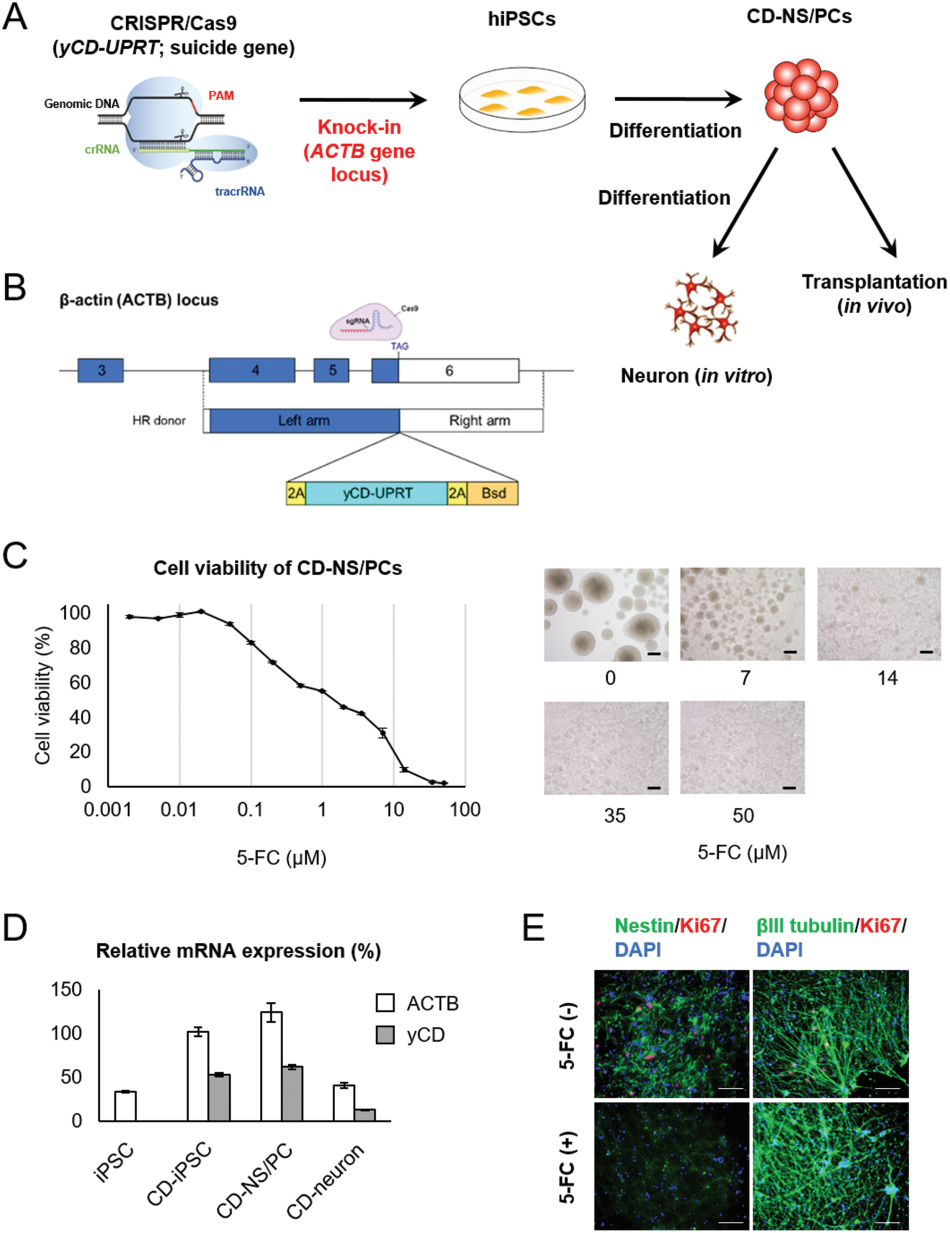
Establishment of CD-NS/PCs by genome editing and differentiation (**A**) schematic overview of genome editing. Some CD-NS/PCs were differentiated into neurons in vitro and subsequently utilized to assess the impact of 5-FC on neuronal cells. (**B**) Schematic depiction of the CRISPR/Cas9-mediated strategy for inserting the yCD-UPRT gene into the *ACTB* locus. Single guide RNA (sgRNA) target sequence and HR donor constructs are shown. (**C**) Cell viability of CD-NS/PCs at each 5-FC concentration. Micrographs of CD-NS/PCs cultured in the presence of 0, 7, 14, 35, or 50 μM of 5-FC for 7 days are also shown. Scale bar, 200 µm. (**D**) Relative mRNA expression of iPSCs, NS/PCs and neuron. (**E**) In vitro analysis of the effect of 5-FC on neurons. Nestin or beta III tubulin is labeled in green; Ki67 in red; and DAPI in blue. Ki67-positive cells remained without 5-FC, while they were absent in the presence of 5-FC, with beta III tubulin-positive neurons being preserved. Scale bar, 100 µm.

1210B2-hiPSCs were derived from human peripheral blood mononuclear cells of a healthy 29-year-old African-American female (Cellular Technology Limited, Shaker Heights, OH, USA). 1210B2-hiPSCs (kindly provided by Shinya Yamanaka, Kyoto Univ., Kyoto, Japan) were cultured with a feeder-free protocol.^15,19^Fig. 1A shows a schematic overview of the experimental design to establish cytosine deaminase-expressing NS/PCs (CD-NS/PCs). The Cas9/sgRNA expression plasmid, pU6-ACTBgRNA-Cas9, was constructed by cloning DNA oligonucleotides coding for sgRNA targeting near the stop codon of *ACTB* into the *BbsI* site of the pX330-U6-Chimeric_BB-CBh-hSpCas9 plasmid.^20^ To generate homologous recombination (HR) donor plasmids, 1-kb fragments of the left and right homology arms of *ACTB* were PCR-amplified from genomic DNA isolated from human fibroblasts (NB1RGB; RIKEN BRC, Ibaraki, Japan) and cloned into the PrecisionX HR donor vector HR100PA-1 (System Biosciences, Palo Alto, CA, USA). Next, a polycistronic cassette containing yeast cytosine deaminase-uracil phosphoribosyl transferase (yCD-UPRT) and a blasticidin-resistance fusion gene (*Bsd*) was inserted between the left and right homology arms, resulting in the HR donor plasmid HR-ACTB-2A-yCD-UPRT-2A-Bsd. HR donor plasmids of *ACTB* were designed to be in frame with the C-terminus of *ACTB* and express fusion proteins joined with a self-cleaving 2A peptide sequence. The PAM sites of ACTBgRNA were substituted by 5ʹ-NTG-3ʹ in HR donor plasmids (Fig. 1B). All plasmids were verified by DNA sequencing.

For transfection, 1 × 10^6^ iPSCs suspended in 100 μL of Opti-MEM (Thermo Fisher Scientific, Waltham, MA, USA) were mixed with pU6-ACTBgRNA-Cas9 (3 μg) and the HR donor plasmid (10 μg), and subjected to electroporation at 125 V for 5 ms using a NEPA21 electroporator (Nepa Gene, Chiba, Japan). Immediately after electroporation, cells were plated in a complete medium and subjected to Bsd selection (0.5 μg/mL). To verify integration, genomic PCR analysis was performed.

### Differentiation into CD-NS/PCs

Embryoid body (EB) formation and NS/PCs generation were performed as previously described. An EB formation method using dual SMAD inhibitors was used. iPSCs were cultured in StemFit AK02N medium (Ajinomoto, Tokyo, Japan) in a 5% CO_2_ incubator. ROCK inhibitor (Fujifilm, Osaka, Japan; Y-27632) was added at the time of cell passaging at a final concentration of 10 µM. To induce EB formation, dual SMAD inhibitors, namely, SB431542 (MedChemExpress, Monmouth Junction, NJ, USA) and LDN193189 (MedChemExpress) were added to StemFit Basic02 medium (Ajinomoto) at final concentrations of 100 nM and 10 µM, respectively. With this medium, the cells were seeded into V bottom 96-well plates at a concentration of 9000 cells/75 µL/well, and cultured for 14 days. On day 14, the aggregates were dissociated to generate the first passage of NS/PCs. The NS/PCs were maintained by culturing them as floating neurospheres in the medium, which consisted of D-MEM/Ham’s F-12 (Fujifilm; 048-29785) with B27 (Thermo Fisher Scientific; 17504044), epidermal growth factor (PeproTech, Cranbury, NJ, USA; AF-100-15), fibroblast growth factor (PeproTech; 100-18B), and leukemia inhibitory factor (Nacalai Tesque, Kyoto, Japan; 07690-31). Following the thawing of the cryopreserved stock, NS/PCs were maintained in culture for a total duration of 25 days, undergoing 3 passages to reach passage 10. Subsequently, cell transplantation was carried out. We dissociated the CD-NS/PC spheres into single cells immediately prior to transplantation.

### Lentiviral Vector-Mediated Transduction

CD-NS/PCs were transduced with the lentiviral vector CSII-EF-ffLuc containing the ffLuc gene (a Venus fluorescent protein^21^ and firefly luciferase fusion gene) at a multiplicity of infection of 2. Transduced cells were seeded as single cells into a 96-well plate and expanded. Single-cell clones stably expressing ffLuc were established.

### Cell Viability Assay

A cell viability assay was performed to evaluate the sensitivity of each CD-NS/PC clone to 5-fluorocytosine (5-FC; Sigma-Aldrich, St. Louis, MO, USA). The assay was performed three days after cells were exposed to 5-FC using a Cell Counting Kit-8 (Dojindo Molecular Technologies, Kumamoto, Japan) as previously described.^15^ The dosages ranged from 0.002 to 50 µM. To account for background absorbance, the absorbance value of the medium at 450 nm was subtracted from each sample. Cell viability was determined by calculating the relative changes in cells that were not exposed to 5-FC. Each experiment was performed in triplicate.

### Analysis of Gene Expression

Total RNA was extracted from NS/PCs or neurons using ISOGEN (NIPPON GENE, Tokyo, Japan). Subsequently, cDNA libraries were prepared from the total RNA by employing ReverTra Ace qPCR RT Kit (TOYOBO, Osaka, Japan). Real-time quantitative PCR (RT-qPCR) was employed to detect the gene expression of GAPDH, ACTB, and yCD. ACTB and yCD expression levels were assessed by calculating the relative changes in comparison with GAPDH through the ΔΔCt method. The RT-qPCR was conducted under the following conditions: 95 °C for 120 s, succeeded by 40 cycles of 95 °C for 15 s, 55°C for 15 s, 72°C for 60 s, and melting curve analysis using Fast SYBR Green Master Mix (Thermo Fisher Scientific) in ViiA 7 Real Time PCR System (Thermo Fisher Scientific). The amplification efficiency of each primer pair (Supplementary Table S1) was evaluated by the standard curve method. Each sample was tested in triplicate.

### In vitro Analysis of the Effect of 5-FC on Neurons

Matrigel (Corning, Corning, NY, USA) and Neurobasal Medium (Thermo Fisher Scientific) containing B27 supplement (Thermo Fisher Scientific) and GlutaMAX supplement (Thermo Fisher Scientific) were mixed at 1:100. Matrix coating was performed using 300 µL of this mixture, and NS/PCs were incubated for a day. The supernatant was then removed, and NS/PCs were incubated for 14 days in Neurobasal Medium containing 0.05 µL of DAPT (Fujifilm) per mL. Half of the medium was changed every 4 days. Subsequently, 5-FC was administered for 6 days. Cells were fluorescently stained with anti-Nestin (1:200, mouse IgG; Chemicon, Tokyo, Japan; MAB5326), anti-Ki67 (1:500, rabbit IgG; Novocastra, Newcastle, UK; NCL-Ki67p), and anti-beta III tubulin (1:200, mouse IgG2a; Sigma-Aldrich; T8660) antibodies.

### Reanalysis of Published RNA-seq Data

Published RNA-seq data for NS/PCs and MSCs were reanalyzed.^18^ We obtained FASTQ data from the NCBI Gene Expression Omnibus (GEO: GSE150470). Raw FASTQ files were trimmed for adapters by Cutadapt^22^ and Salmon^23^ to generate the transcripts per million (TPM) and estimated counts using the transcript index from GRCh38 (Gencode v40). We selected genes associated with terms “neurotrophic,” “neuro growth,” and “neurotrophin” in Gene Ontology. Heatmaps of gene expression were drawn on the row-wise *z*-value of log2(TPM + 1) for each gene.

### Animal Model of TBI

**Figure 2.**
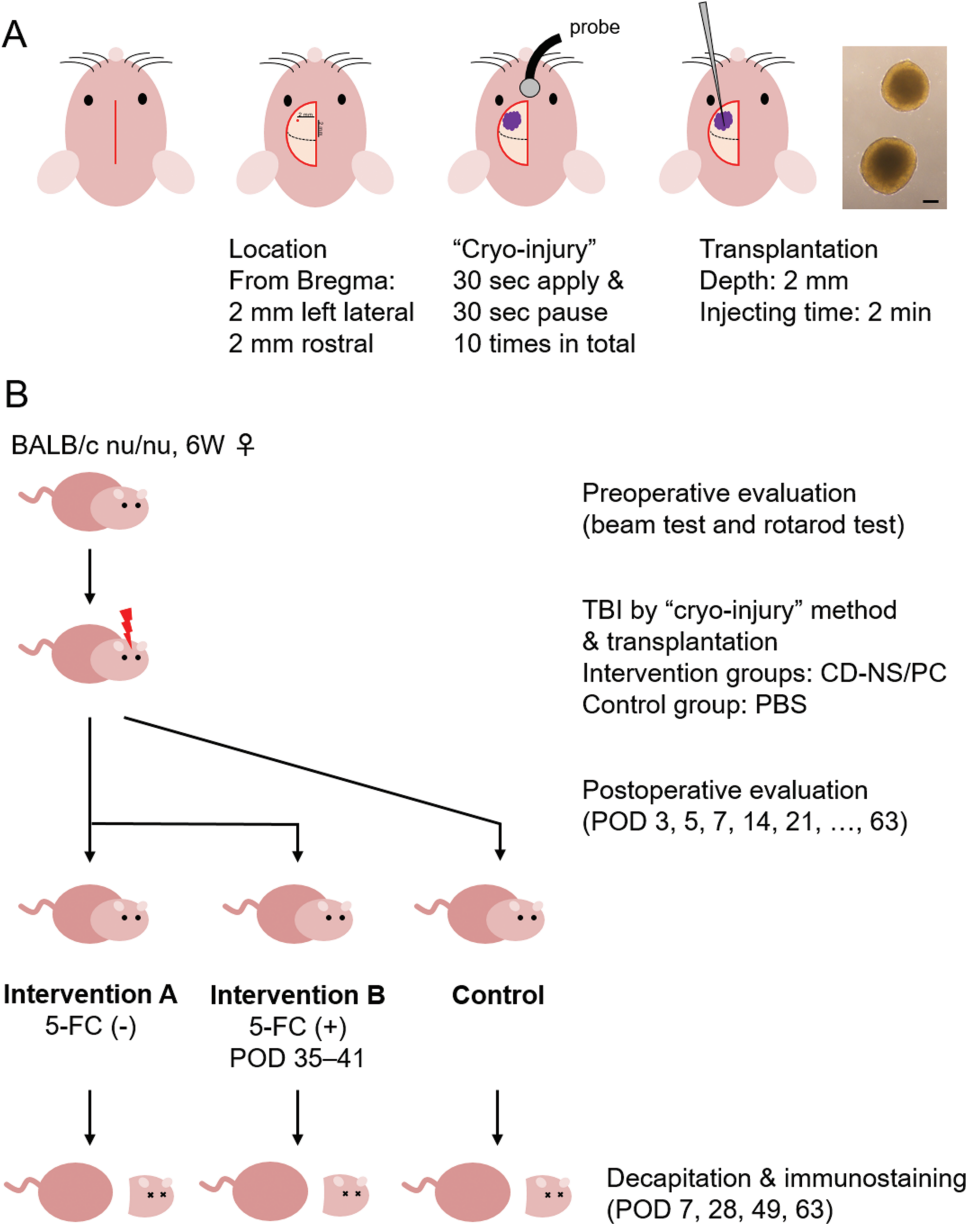
Schematic overview of the experiment (**A**) sequential illustrations showing overall surgical procedures of the TBI mouse model, including the information on skin incision, contusion location, “cryo-injury” method, and transplantation technique. A micrograph of transplanted NS/PCs is also presented. Scale bar, 200 µm. (**B**) Schema of the experimental time course, including the timing of behavioral evaluation and decapitation. Note that 5-FC was consecutively administered from postoperative days 35 to 48 in intervention group B. POD, postoperative day.

All experiments were performed by Guidelines for the Care and Use of Laboratory Animals of Keio University (Approval No. 14057) and Guide for the Care and Use of Laboratory Animals (National Institutes of Health, Bethesda, MD, USA). The brain injury model was created based on a previously reported “cryo-injury” method.^24^ Six-week-old female BALB/c nu/nu mice (Sankyo Labo Service Corporation, Tokyo, Japan) were housed at a constant temperature with a 12-h light-dark cycle. Mice were anesthetized with an intraperitoneal injection of 200 μL of mixed anesthetic (0.3 mg medetomidine, 4 mg midazolam, and 5 mg butorphanol in 10 mL of normal saline) and fixed in a stereotaxic frame. For each mouse, a longitudinal skin incision was made on the head, and a burr hole was drilled out on the cranium (Emax EVOlution; Nakanishi, Tochigi, Japan) at a point 2 mm anterior and 2 mm left lateral to the bregma (motor-sensory cortex). Next, a metal probe chilled with CO_2_ gas (Cryomatic Console MKII; Keeler, Malvern, PA, USA) was applied to the brain surface (Fig. 2A). Ten sessions were performed, each of which comprising a 30-s probe application followed by a 30-s break, as previously described.^24^ Following intracerebral injection of NS/PC solution (intervention groups) or phosphate-buffered saline (PBS, control group) at the contusion site, the skin was closed with surgical staples. These procedures were microscopically conducted. Mice were returned to their cages and allowed free access to food and water, with no antibiotics given.

### Study Design and Cell Transplantation

Preceding the main experimental framework, we performed 2 preliminary experiments. The first involved tracking the movement of CD-NS/PCs in 3 mice, whereas the second evaluated the permeability of the blood-brain barrier through Evans blue leakage observed in eight mice. The specific procedures will be elaborated upon later.

Subsequently, we proceeded to assess the motor function and immunohistochemical findings following in vivo transplantation of CD-NS/PCs. A total of 28 mice were divided into 3 experimental groups (Fig. 2B): (1) control group receiving PBS, (2) intervention group A receiving CD-NS/PC alone, and (3) intervention group B receiving CD-NS/PC and intraperitoneal 5-FC for 14 consecutive days from postoperative day 35 to eliminate undifferentiated CD-NS/PCs by the suicide gene (yCD-UPRT). In the intervention groups, 4 µL of CD-NS/PC solution (containing 5 × 10^5^ single cells) was stereotaxically injected using a 10-μL Hamilton syringe (Hamilton Company, Reno, NV, USA; 701RN; outer diameter 0.47 mm, inner diameter 0.13 mm); ie, 2 mm in depth from the contusion center on the brain surface produced by “cryo-injury” technique. In the control group, 4 µL of PBS was similarly administered. The injection was meticulously performed over 2 min to avoid unexpected solution loss or overflow. The mice underwent behavioral assessments using 2 modalities, namely the beam walking test and accelerating rotarod test, at postoperative days 3, 5, and 7, followed by weekly assessments until day 63. In addition, the mice were sacrificed at predetermined timings and subjected to immunohistochemical evaluation. An overview of the time course is presented in Supplementary Fig. S1.

### Bioluminescent Imaging to Monitor the Migration of CD-NS/PCs

In the first preliminary experiment, a Xenogen-IVIS 100 imaging system (PerkinElmer, Waltham, MA, USA) was used for in vivo bioluminescent imaging (BLI), as described previously.^25,26^ Three mice anesthetized with isoflurane gas were intraperitoneally injected with 300 mg/kg of d-luciferin (VivoGlo Luciferin; Promega, Madison, WI, USA) and placed on a warmed stage inside the camera box of the IVIS imaging system coupled with a charge-coupled device camera. Images were quantified as photons per minute for CD-NS/PCs. To evaluate the migration activity of CD-NS/PCs, BLI was used to monitor CD-NS/PCs in vivo. TBI was made on the anterior-left side, as described above. Next, CD-NS/PCs were transplanted into the contralateral side (right striatum). Histological analysis was performed after sacrificing mice by decapitation on postoperative day 35. Images were quantified as photons per minute for CD-NS/PCs.

### Determination of Blood-Brain Barrier Permeability

The second preliminary experiment comprised 8 mice, with 4 of them receiving CD-NS/PC transplantation into the brain contusion, while the other 4 underwent PBS injection as a control group. On postoperative days 7, 14, and 42, we injected 300 µL of 2% saline solution of Evans blue (Nacalai Tesque) into the caudal veins of mice before decapitation. After the circulation of dye for 2 h, mice were sacrificed and transcardially perfused with 4% paraformaldehyde (PFA). Brain tissues were fixed with 4% PFA followed by soaking in 10% and 20% sucrose at 4 ºC overnight. We macroscopically evaluated differences in blood-brain barrier permeability between control and intervention groups by the degree of Evans blue leakage. In addition, to provide a quantitative assessment, we calculated the area of Evans blue leakage in the photographs on postoperative days 14 and 42.

### Beam Walking Test

The beam walk tests complex motor coordination. The beam apparatus (BT-18; Shin Factory, Fukuoka, Japan) consisted of a 90-cm beam with a flat surface fixed at 90 cm height, and a black box harboring a cage with nesting material at the end of the beam. The width of the beam tapered from 13 to 5 mm toward the end. To attract the mouse, a fan on the box wall blew air in the direction of the beam. The mouse was placed with its nasal apex 80 cm away from the goal, and its tail was released to allow movement. The time it took for the mouse to traverse the beam and reach the black box was recorded. Prior to the initial assessment before surgery, mice were subjected to training twice.

### Accelerating Rotarod Test

The rotarod unit (Acceler Rota-Rod for Mice 7650; Ugo Basile SRL, Gemonio, Italy) was equipped with a 3.0-cm diameter rotating rod and 5 separated compartments to accommodate mice. Initially, the rod was rotating at 2 rpm. Subsequently, the speed was automatically increased at a constant rate from 2 to 30 rpm over 300 s. The time was recorded when the mouse fell off, or its nasal apex came below the rotating axis. In the same way as the beam walking test, mice practiced the preoperative accelerating rotarod test twice before the baseline assessment.

### Immunohistochemical and Cytochemical Analysis

Brain tissue was formalin-fixed and paraffin-embedded, and standard immunohistochemical and cytochemical procedures were carried out. For immunohistochemistry, paraffin-embedded tissue sections (3 µm thick) were initially fluorescently stained with an anti-STEM121 antibody (1:500, mouse IgG; Takara, Kusatsu, Japan; Y40410) or anti-STEM101 antibody (1:100, mouse IgG; Takara; Y40400). Double staining was then performed using one of the following antibodies: anti-GFAP (1:200, rabbit IgG; Abcam, Cambridge, UK; ab33922), anti-NeuN (1:200, mouse IgG; Chemicon; MAB377), anti-Olig2 (1:200, rabbit IgG; Immuno-Biological Laboratories, Fujioka, Japan; 18953), anti-Nestin (1:200, mouse IgG; Chemicon; MAB5326), anti-Nestin (1:100, mouse IgG1; Thermo Fisher Scientific; MA1-110), and anti-Ki67 (1:500, rabbit IgG; Novocastra; NCL-Ki67p). Finally, the third staining was conducted with an anti-cleaved caspase 3 antibody (1:200, rabbit IgG; Cell Signaling Technology, Danvers, MA, USA; 9661). After staining the nuclei of each section with 4ʹ,6-diamidino-2-phenylindole (DAPI), samples were mounted with ProLong Glass Antifade Mountant (Thermo Fisher Scientific). Specimens were examined with an All-In-One Fluorescence Microscope (BZ-9000 Biorevo; Keyence, Osaka, Japan) and a confocal laser scanning microscope (FV3000; Olympus, Tokyo, Japan). The former was utilized to capture comprehensive images at low magnification, while the latter was employed to visualize the merging of fluorescent dyes at high magnification. The proportion of STEM121-positive cells exhibiting dye merge with each marker (NeuN, GFAP, Olig2, and Nestin) in tissue sections was evaluated by quantifying cell counts. The percentage of STEM101-positive cells showing Ki67 positivity was also assessed. Moreover, to evaluate brain atrophy, the area of the left lateral ventricle (square µm) was measured on a histological cross-section containing the contusion center, as well as the area of the non-injured right lateral ventricle.

### Statistical Analysis

To assess differences in behavioral performance between the experimental groups, Student’s *t*-test was performed for times (seconds) recorded in beam walking and accelerating rotarod tests during each evaluation. Additionally, the quantified area manifesting Evans blue extravasation, positive rates for dye merge observed in histological investigation, and the microscopically measured area of bilateral lateral ventricles were also subjected to *t*-test. All analyses were conducted with statistical software (Statcel 3; OMS Publishing, Tokyo, Japan; GraphPad Prism version 8; GraphPad Software, San Diego, CA, USA) and independently repeated twice.

## Results

### Establishment of CD-NS/PCs

The suicide gene (yCD-UPRT) was successfully transduced into the *ACTB* locus of hiPSCs. The Sanger sequencing results verifying successful incorporation of transgene is depicted in Supplementary Fig. S2A. The viability of NS/PCs derived from these genetically modified iPSCs declined in response to elevated 5-FC concentration (Fig. 1C). Expression of yCD-UPRT gene was maintained after differentiation from iPSCs to NS/PCs and neurons (Fig. 1D). Most neurotrophic factor genes showed no significant difference in expression between NS/PCs and CD-NS/PCs (Supplementary Fig. S2B). In vitro analysis demonstrated that the absence of 5-FC resulted in the persistence of Ki67-positive proliferative cells, while they were not observed in the presence of 5-FC (Fig. 1E). Notably, the differentiated neurons with beta III tubulin positivity were not affected by 5-FU.

### Analysis of Migration

We observed that CD-NS/PCs, if transplanted into the contralateral side (right) of the injury site (left), migrated towards the injury site from day 14 (Supplementary Fig. S3A). Immunohistochemical staining of STEM121, reacting specifically with a cytoplasmic protein of transplanted human cells, detected graft-derived CD-NS/PCs on the corpus callosum and contralateral injury site (Supplementary Fig. S3B).

### Macroscopic Evaluation of TBI

**Figure 3.**
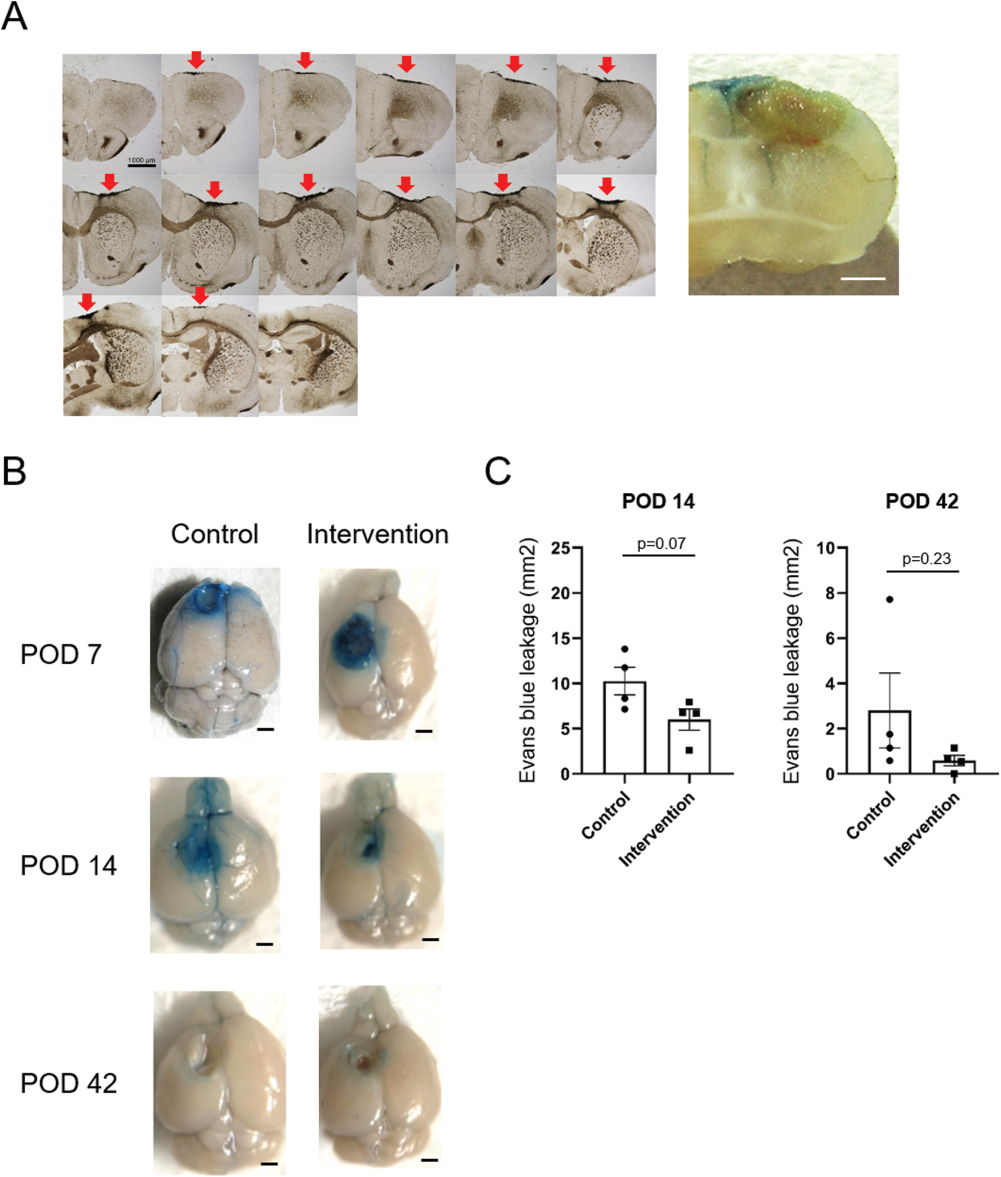
The effect of TBI by “cryo-injury” method (**A**) TBI induced by the “cryo-injury” method was observed on cross-sectional 3,3ʹ-diaminobenzidine staining (left). Arrows indicate the contusion area on the brain surface. At the same time, blood-brain barrier disruption was visualized by intravenous Evans blue (right). Note the deep extension of the contusion. Scale bar, 1000 µm. (**B**) Macroscopic visualization of blood-brain barrier disruption by Evans blue leakage over time. Mice were injected with Evans blue through the caudal vein 2 hours before decapitation. Prolonged dye leakage was observed in the control group. Scale bar, 1000 µm. POD, postoperative day. (**C**) Quantitative assessment of Evans blue extravasation. The extent of dye leakage was comparably lesser in the intervention group on postoperative days 14 and 42.

The effect of TBI induced by the “cryo-injury” method was observed to reach as deep as the external capsule on serial cross-section. Additionally, disruption of the blood-brain barrier was macroscopically visualized by Evans blue, suggesting that the brain contusion was not only limited to the surface but extended to the deep white matter (Fig. 3A). In particular, the control group showed a tendency toward prolonged dye leakage, which was especially pronounced on postoperative day 14 (Fig. 3B). On quantitative evaluation, a trend toward smaller area of Evans blue leakage was also noted in the intervention group on postoperative days 14 and 42, although statistical significance was not observed (*P* = .07 and .23, respectively) (Fig. 3C).

### Behavioral Assessment

**Figure 4.**
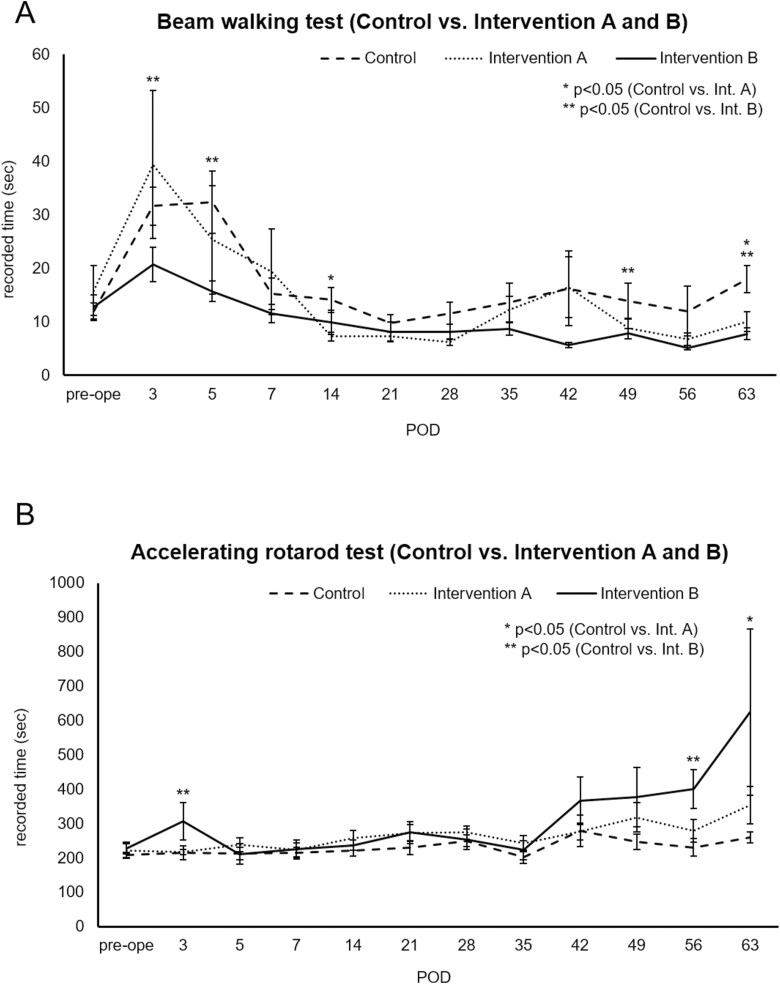
Results of behavioral assessment with each modality (**A**) Graph comparing beam walking test performance (mean seconds) over time for control and intervention groups. (**B**) Graph comparing accelerating rotarod test performance (mean seconds) over time for control and intervention groups. Asterisks and double asterisks indicate the time point when a significant difference was observed between the 2 groups (control and intervention A or control and intervention B, respectively). POD, postoperative day.

Beam walking test results were better (ie, recorded times were shorter) for intervention group B (NS/PCs plus 5-FC) compared with the control group over the entire observation period (Fig. 4A). In particular, significant differences were observed in postoperative days 3, 5, 49, and 63 (*P* = .02, .01, .04, and .002, respectively). Almost the same tendency was noted for intervention group A. Accelerating rotarod test performance also tended to improve (ie, recorded times became longer) in the intervention groups as TBI entered the chronic phase, with significant differences at some time points (Fig. 4B). Importantly, no significant differences were observed between intervention groups A and B for the rotarod test. It should be noted that one specific mouse belonging to intervention group A rarely scored less than 10 seconds in the beam walking test (including preoperative training), meaning its recorded time always exceeded the average of the same group. Data from this mouse were excluded from the analysis of the beam walking test.

### Histological Investigation

**Figure 5.**
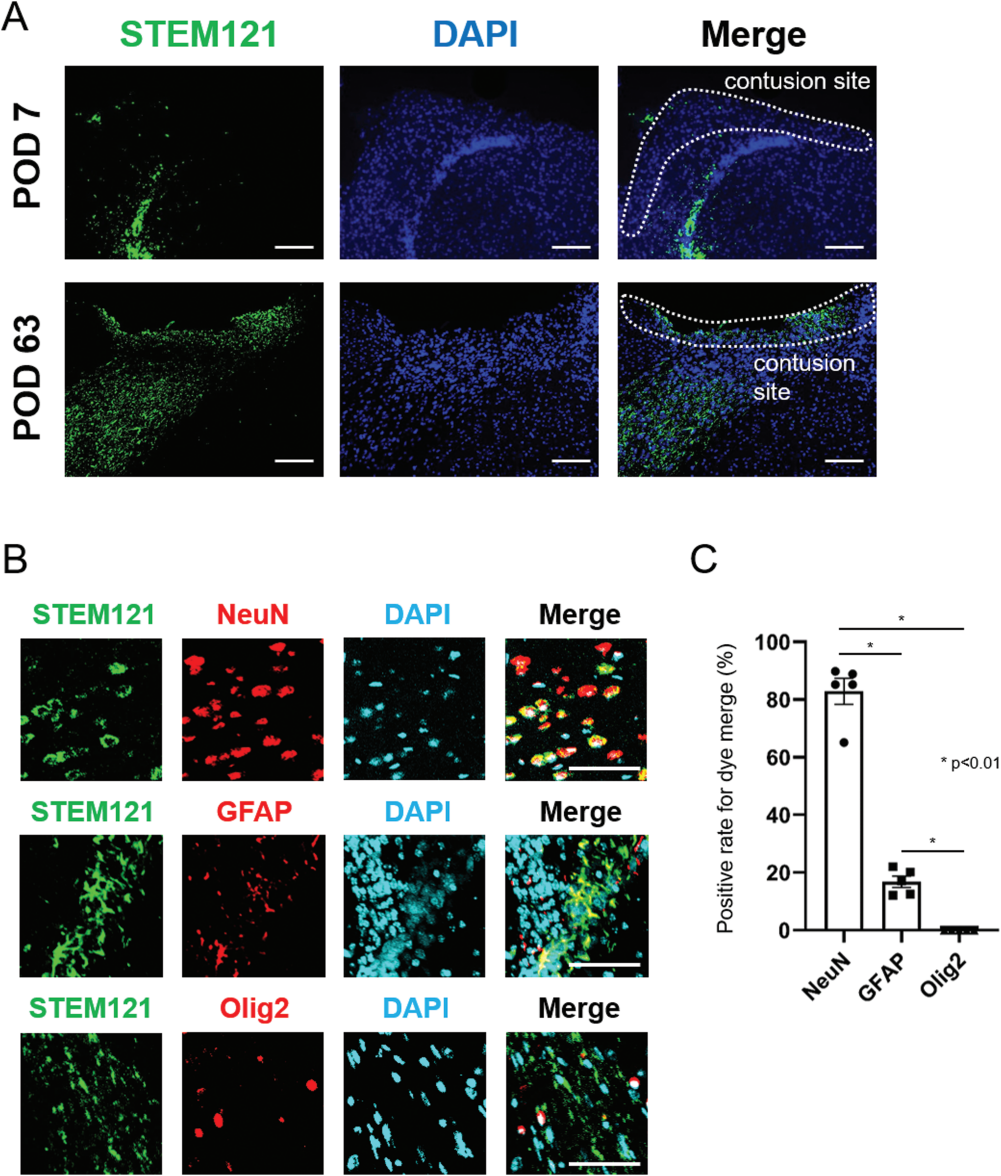
Histopathological findings after transplantation of CD-NS/PCs multiple fluorescent staining of brains transplanted with NS/PCs (intervention group A). STEM121 is labeled in green; DAPI in blue; and NeuN, GFAP, and Olig2 are in red. (**A**) Migration of STEM121-positive NS/PCs to the contusion site over 9 weeks after injury. Scale bar, 100 µm. (**B**) Magnified images of the contusion site. STEM121-positive NS/PCs displayed the most robust dye merge with NeuN (83%), whereas only a few merged with GFAP or Olig2 (17% or 0%, respectively). Scale bar, 50 µm. (**C**) Positive rate for co-localization of STEM121 with NeuN, GFAP or Olig2.

**Figure 6.**
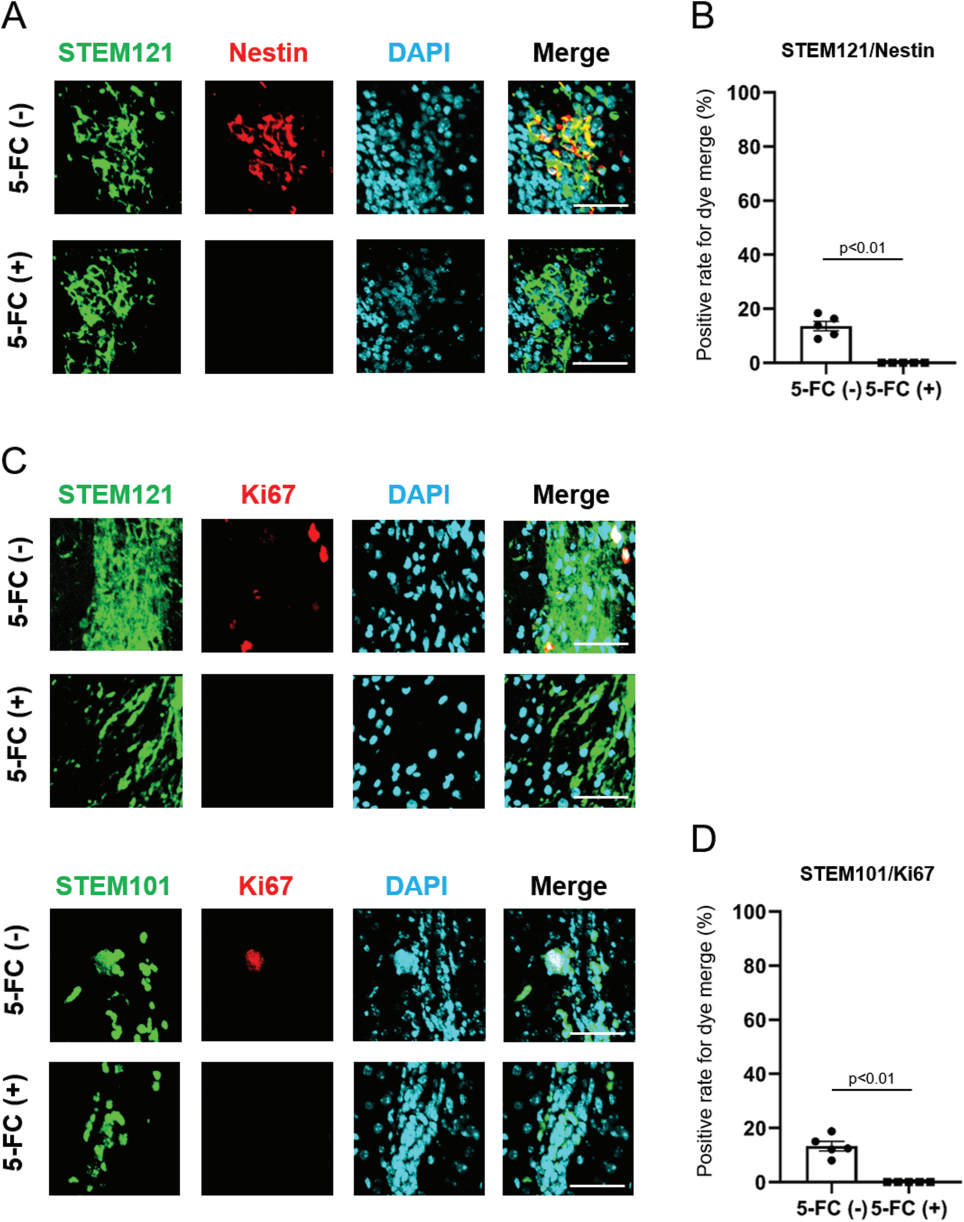
The effect of 5-FC administration on transplanted CD-NS/PCs comparison between intervention groups A (without 5-FC) and B (with 5-FC) on postoperative day 63. STEM121 or STEM101 is labeled in green; DAPI in blue; and Nestin or Ki67 in red. (**A**) Undifferentiated Nestin-positive cells remained in small numbers in intervention group A (14%), while such cells were not observed in intervention group B. Scale bar, 50 µm. (**B**) Positive rate for colocalization of STEM121 with Nestin. (**C**) For both STEM121 and STEM101, cells with Ki67 positivity remained in intervention group A (13%), while they were not detected in intervention group B. Scale bar, 50 µm. (**D**) Positive rate for dye merge of STEM101 and Ki67.

Microscopically, a small number of NS/PCs reached the contusion site on postoperative day 7. A large number of NS/PCs were observed migrating to the lesion on day 63 (Fig. 5A). Multiple fluorescent staining performed on day 63 revealed that STEM121-positive NS/PCs had the strongest overlap with NeuN (Fig. 5B), while only a few overlapped with GFAP. No NS/PCs positive for Olig2 and STEM121 staining were observed. This finding was quantified by cell counting (Fig. 5C). Around 80% of cells exhibited merge of NeuN and STEM121, whereas, for GFAP and Olig2, the co-localization was detected in 17% and 0% of cells, respectively. Furthermore, histology on postoperative day 63 demonstrated that 14% of cells in intervention group A (without 5-FC) proved to be Nestin positive (ie, undifferentiated), while such cells were absent in intervention group B (with 5-FC) (Fig. 6A, 6B). Additionally, Ki67-positive cells persisted in the non-5-FC-treated group (13%), whereas they were not detected in the 5-FC-treated group, suggesting that proliferative STEM121 or STEM101-positive cells disappeared in the presence of 5-FC (Fig. 6C, 6D). These differences in cell counts were found to be statistically significant at a *P*-value of < .01 for all groups.

**Figure 7.**
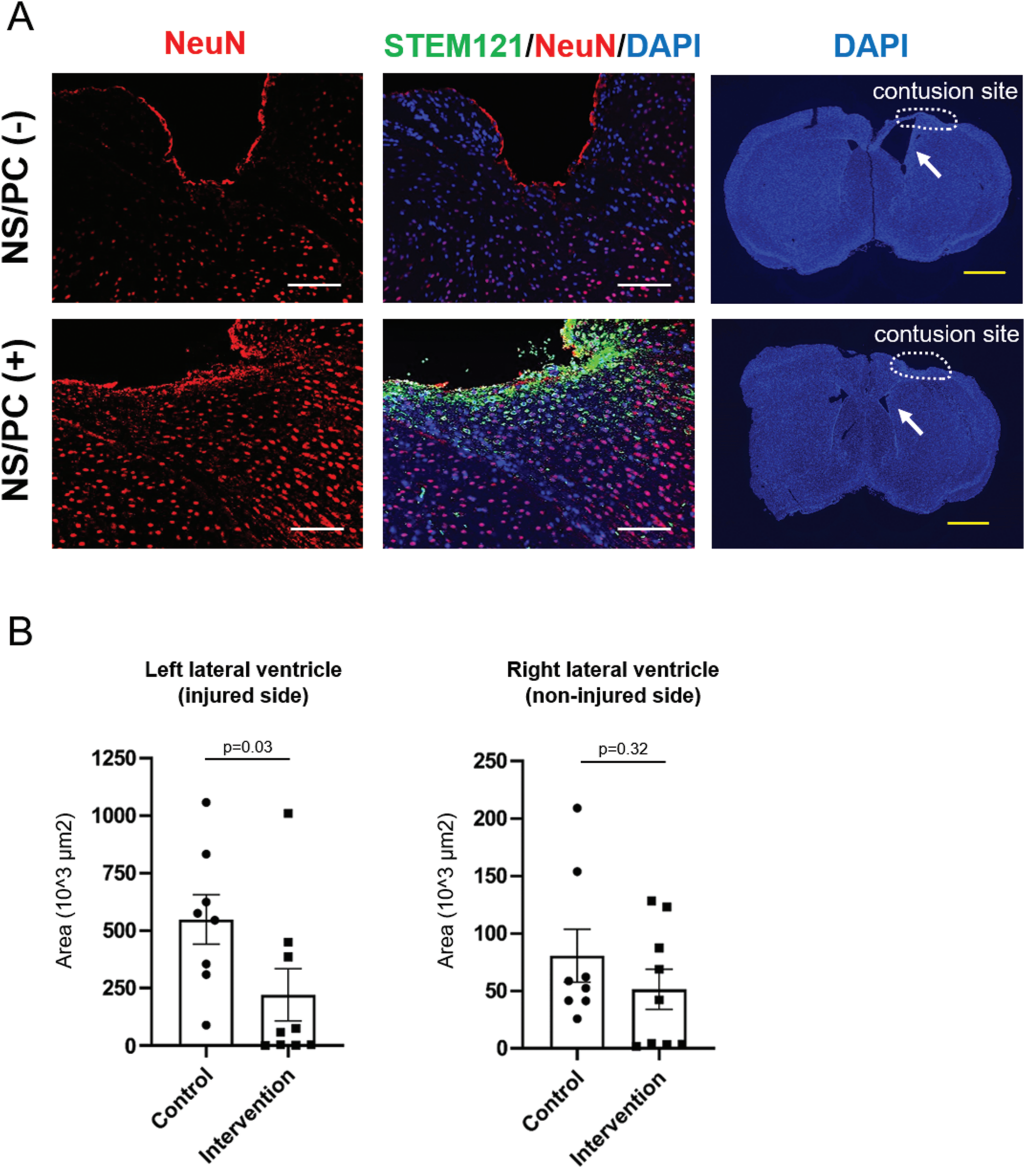
Prevention of cerebral atrophy and lateral ventricle enlargement after TBI Multiple fluorescent staining of brains transplanted with NS/PCs. STEM121 is labeled in green, DAPI in blue, and NeuN in red. (**A**) Comparison between the control group (without NS/PC transplantation, upper row) and intervention group A (with NS/PC transplantation, lower row) on day 63. Extensive loss of NeuN-positive neurons around the contusion site in the control group is shown. Note that STEM121-positive NS/PCs aggregate near the contusion site in intervention group A. Left lateral ventricle (arrows) was comparably enlarged without NS/PC transplantation. White scale bar, 100 µm; yellow scale bar, 1000 µm. (**B**) The cross-sectional measured area of the left or right lateral ventricle on postoperative day 63. The control group had significantly larger left ventricular areas than both intervention groups, whereas no significant difference was observed for the right lateral ventricle (non-injured side).

Evaluating the effect of NS/PC transplantation revealed extensive loss of the original NeuN-positive neurons around the contusion site on postoperative day 63 in the control (non-transplanted) group, while these cells appeared to be spared in intervention group A (Fig. 7A). Additionally, the cross-sectional measured area of the left lateral ventricle was significantly more prominent in the control group compared with both intervention groups (Fig. 7B). In contrast, the area of the right lateral ventricle (non-injured side) exhibited no significant differences between the two experimental cohorts. The average area was smaller in comparison to that on the injured side.

## Discussion

The present study demonstrated the migration of NS/PCs to the brain contusion site in vivo and successful differentiation into neurons during the chronic phase. In addition, significant functional improvements in motor skills and prevention of brain atrophy were noted in mice transplanted with NS/PCs. Furthermore, 5-FC administration resulted in the complete disappearance of undifferentiated NS/PCs, whereas differentiated and viable transplanted cells were preserved. These findings suggest that genome-edited hiPSC-derived NS/PCs can overcome the general concern of tumorigenesis and show promise for safe regenerative medicine.

Transduction via lentiviral vectors integrates randomly into the host genome, raising concerns about insertional mutagenesis and oncogene activation. Transgene silencing is frequently observed in hiPSCs. Furthermore, stable constitutive expression of therapeutic genes in hiPSCs is difficult using viral vectors.^16,17^ To address this problem, the yCD-UPRT gene was inserted into the *ACTB* housekeeping gene locus via CRISPR/Cas9 to achieve stable and constant expression of yCD-UPRT in hiPSCs.^18^

We assume that the initial neuroprotective effects against TBI observed in this study are associated with the secretion of various neurotrophic factors from NS/PCs during the acute phase. Reanalysis of published RNA-seq data indicated that gene integration into the *ACTB* locus does not affect the gene expression of most neurotrophic factors. Importantly, we discovered that there is no substantial disparity in gene expression between genetically modified and non-genetically modified iPSC-derived NS/PCs. However, it remains a topic for future investigation to ascertain which factors are actually present and exerting the most significant impact.

During the chronic phase, NS/PCs have the potential to provide neuroprotection and reconstruct neural networks. Interestingly, ipsilateral ventriculomegaly was significantly less evident in the intervention groups compared with the control group. In a previous study using MSCs, hydrocephalus after severe intraventricular hemorrhage was reportedly prevented due to the anti-inflammatory effects of MSCs, rather than their regenerative capabilities.^27^ However, NS/PCs completing migration during the chronic phase were viable as neurons to suppress brain atrophy—a regenerative effect that is expected to improve higher brain function.

Most importantly, the safety of our treatment strategy was demonstrated. After 5-FC administration, 5-fluorouracil (5-FU) converted from 5-FC by cytosine deaminase selectively eliminated redundant undifferentiated transplanted NS/PCs, preserving the adjacent neuronal structures. Indeed, no significant differences in behavioral assessments were noted between intervention groups with or without 5-FC administration. Transplanted NS/PCs terminally differentiated into NeuN-positive neurons in vivo and displayed no further proliferative ability. Additionally, our initial in vitro investigations have demonstrated that the expression of yCD-UPRT is reduced in differentiated neurons, presumably resulting in decreased release of 5-FU. It has also been observed that the neurons are not affected by 5-FU, most likely due to their incapacity to proliferate.

Further work is needed to optimize the appropriate transplantation site and timing for this cell-based gene therapy. The injury site was the main stereotactic injection target in the present study. Wallenquist et al. reported higher survival rates of NS/PCs transplanted into the lateral ventricle.^28^ Future studies should compare the effect of different transplantation sites. However, as indicated above, NS/PCs in this study were so migratory that subtle differences in the transplantation site might not be a significant problem. Besides the transplantation site, when to perform cell-based therapy is a considerable concern. In a spinal cord injury model, functional improvement by neural progenitor cell transplantation was more remarkable in the acute phase than in the subacute or chronic phases.^29^ A phase III study in patients with chronic spinal cord injury also suggested that injection of MSCs into the intramedullary and subdural spaces resulted in a weak therapeutic effect in only two of 16 patients.^30^ However, as discussed above, NS/PCs have the potential for both reconstruction of neural networks and neuroprotection. In future research, the effect of iPSC-derived NS/PC-mediated cell-based therapy should be examined for chronic TBI.

A major limitation to the engraftment of CD-NS/PCs is the lack of host innate immune responses in immunodeficient mice. TBI has been shown to induce immune-mediated inflammatory reactions that can last post-injury.^31^ The immune system plays a role in driving the secondary phase of tissue damage following TBI. The possible influence of NS/PCs on the immune system should also be mentioned. While findings from stroke studies have shown that transplanted NS/PCs can suppress T-cell responses and down-regulate inflammation,^32,33^ the effects of immunomodulation by transplanted cells can be either beneficial or deleterious, and not always advantageous.^34^ It has been noted that the by-stander effect of transplanted cells may impact mature neurons, particularly in hosts with normal immune systems.^35^ Therefore, while speculative due to the lack of data, it is possible that undifferentiated cells dying after 5-FC administration may induce an excessive immune response in hosts with normal immune systems, potentially leading to secondary brain damage that could counteract the neuroprotective effects of NS/PCs. The use of immunocompromised nude mice in this study remains a constraint. Further studies are warranted to evaluate this treatment strategy’s effect on the microenvironment of TBI.

## Conclusion

iPSC-derived NS/PC transplantation during the acute phase of TBI effectively improved motor function and prevented brain atrophy during the chronic phase. Furthermore, suicide gene transfer solved the concern for tumorigenesis of transplanted cells. Thus, genome-edited iPSC-derived NS/PCs can be applied as a safe regenerative medicine for TBI.

## Supplementary Material

sxad028_suppl_Supplementary_Figure_S1

sxad028_suppl_Supplementary_Figure_S2

sxad028_suppl_Supplementary_Figure_S3

sxad028_suppl_Supplementary_Table_S1

## Data Availability

RNA-seq data have been deposited in the NCBI Gene Expression Omnibus (GEO) under accession number GSE150470. All other data in this article are available from the corresponding author upon reasonable request.
