# Supplementary figures and images for: Neuroprotective Effects of Genome-Edited Human iPS Cell-Derived Neural Stem/Progenitor Cells on Traumatic Brain Injury
Source: Stem Cells. 2023 Apr 8;41(6):603–16. doi: 10.1093/stmcls/sxad028 (PMC10267696; doi:10.1093/stmcls/sxad028)

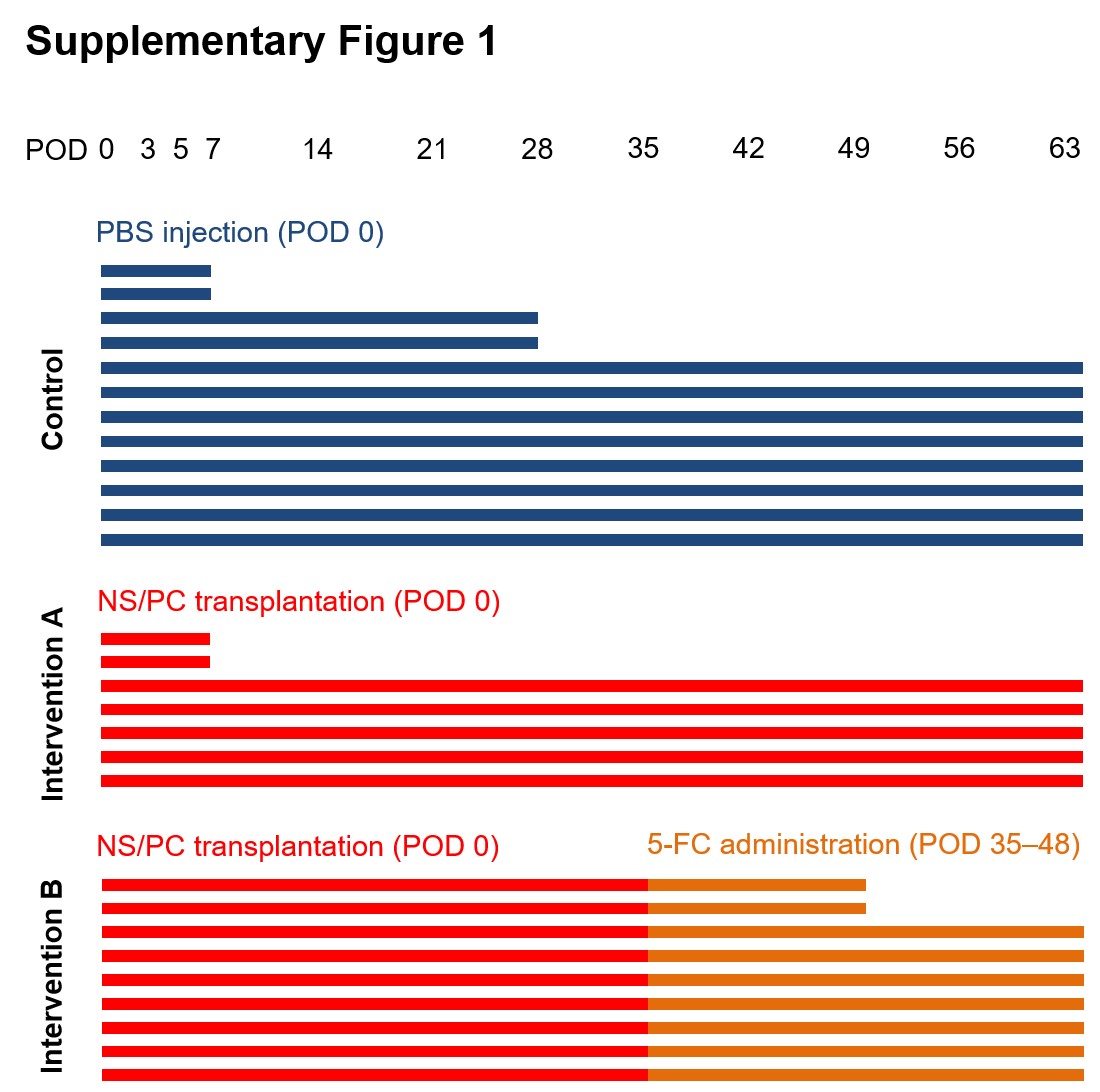

Supplement: sxad028_suppl_Supplementary_Figure_S1 [file sxad028_suppl_supplementary_figure_s1.jpeg]

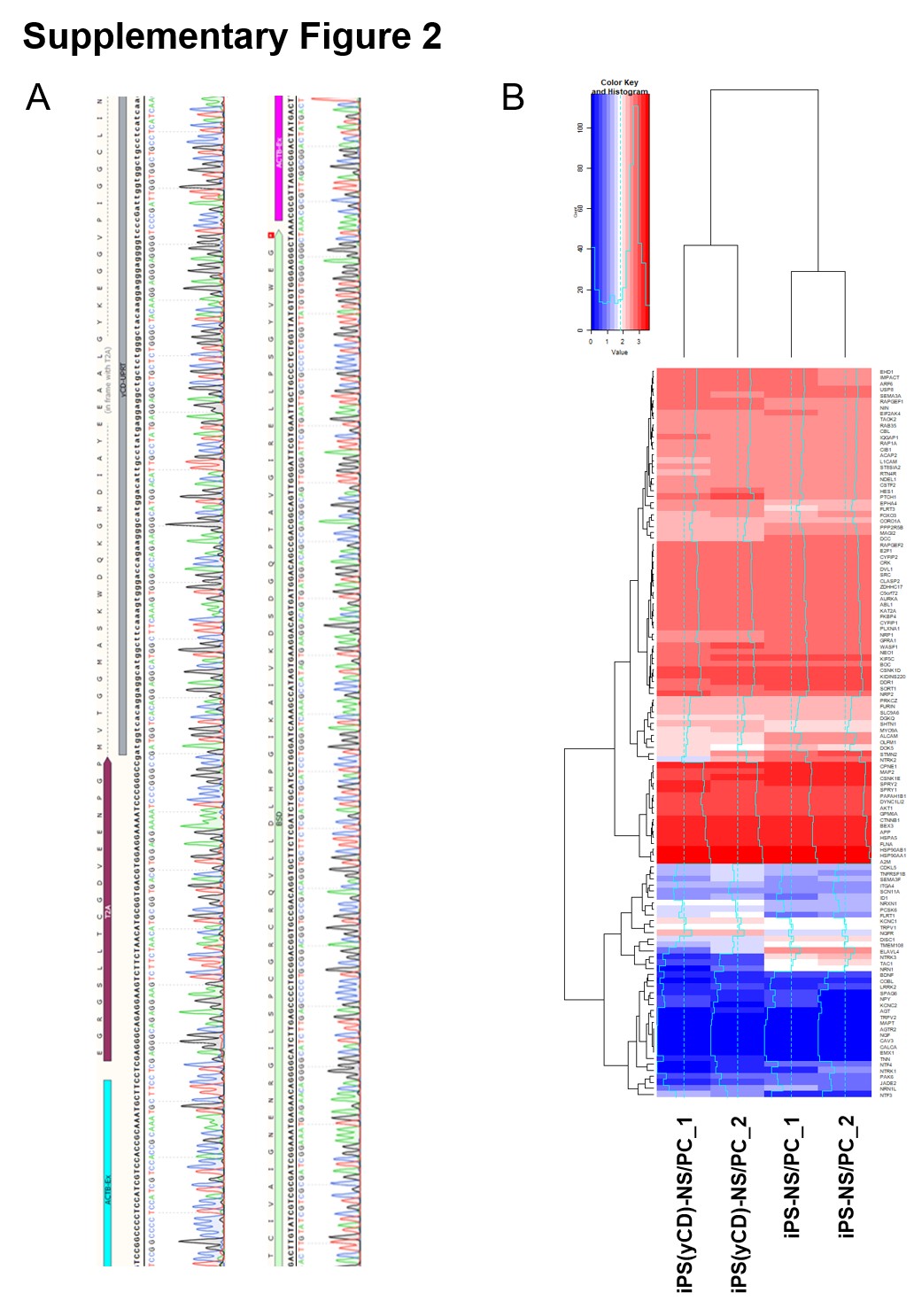

Supplement: sxad028_suppl_Supplementary_Figure_S2 [file sxad028_suppl_supplementary_figure_s2.jpeg]

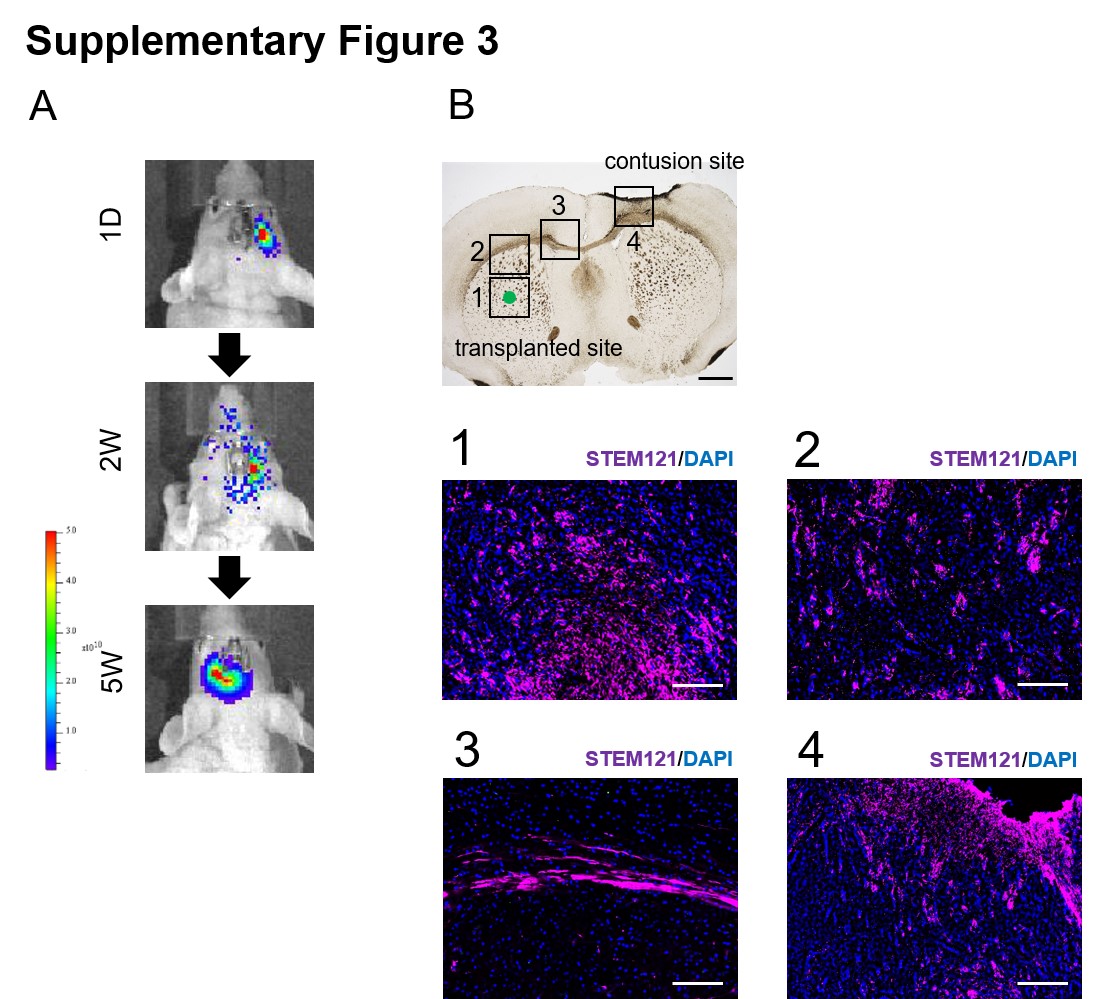

Supplement: sxad028_suppl_Supplementary_Figure_S3 [file sxad028_suppl_supplementary_figure_s3.jpeg]
