# Supplementary material for: Neuroprotective Effects of Genome-Edited Human iPS Cell-Derived Neural Stem/Progenitor Cells on Traumatic Brain Injury
Source: Stem Cells. 2023 Apr 8;41(6):603–16. doi: 10.1093/stmcls/sxad028 (PMC10267696; doi:10.1093/stmcls/sxad028)
Supplement: sxad028_suppl_Supplementary_Table_S1 [file sxad028_suppl_supplementary_table_s1.docx]

**Supplementary Table 1**

| GAPDH-Forward | *GTCCACTGGCGTCTTCACCA* |
| --- | --- |
| GAPDH-Reverse | *GTGGCAGTGATGGCATGGAC* |
| ACTB-Forward | *GATCAAGATCATTGCTCCTCCT* |
| ACTB-Reverse | *GGGTGTAACGCAACTAAGTCA* |
| yCD-Forward | *CACCATGGTCACAGGAGGCAT* |
| yCD-Reverse | *TTAGACACAGTAGTATCTGTC* |
